# Complete resection of the alar folds in eight standing horses with a bipolar dividing and vessel‐sealing device

**DOI:** 10.1111/vsu.13383

**Published:** 2020-02-06

**Authors:** Airina Kallmyr, Ellen M. Giving, Lars O. Moen, Marianne Øverlie, Therese Holm, Florent David

**Affiliations:** ^1^ Bjerke Dyrehospital‐En Rikstotoklinikk Oslo Norway; ^2^ Romerike Hesteklinikk AS Kjeller Norway; ^3^ Evidensia Lørenskog Dyreklinikk Lørenskog Norway; ^4^ Equine Veterinary Medical Center a member of Qatar Foundation, Doha Qatar; ^5^ College of Health & Life Science Hamad Bin Khalifa University Doha Qatar

## Abstract

**Objective:**

To describe a resection technique of the alar folds in the standing horse.

**Study design:**

Retrospective case study.

**Animals:**

Eight Standardbred racing trotters.

**Methods:**

Horses in which alar fold collapse had been diagnosed between 2017 and 2018 were included in this study. All horses underwent alar fold resection under standing sedation and regional anesthesia with a bipolar electrosurgical open sealer/divider device (LigaSure). Intraoperative and postoperative complications were recorded. A Wilcoxon signed‐rank test was used to compare differences in median prize money earning pre‐surgery and post‐surgery (*P* < .05).

**Results:**

The surgical procedure was short (20‐30 min), with minimal (1/8) to no (7/8) bleeding and was well tolerated in all cases. Complete resection of the alar folds along with 3 to 5 cm of the ventral conchal cartilage was achieved. No complications were observed post‐surgery with satisfactory second intention healing, allowing return to training/racing within 3 to 6 weeks post‐surgery in all cases. Median earnings post‐surgery increased (*P* = .03) compared with pre‐surgery.

**Conclusion:**

Alar fold resection with bipolar electrosurgical energy offered a good alternative to the traditional surgical approaches performed under general anesthesia. The surgery significantly improved race earnings and performance while avoiding the risk associated with general anesthesia and offered a short and complication‐free rehabilitation period.

**Clinical impact:**

This study describes a surgical technique offering a novel approach to resection of the alar folds in the standing horse.

## 1 INTRODUCTION

Alar folds (AF) are mucocutaneous folds located dorsorostrally in the nasal cavities, creating a divide between the true nostril and a smaller upper diverticulum known as the false nostril. The alar cartilage, an extension of the ventral conchal cartilage, is attached/part of the AF caudally.^1^ The alar cartilage is elevated when the *transversus nasi* muscle contracts during deep inspiration, which results in a closing of the opening to the false nostril.^2^

Alar fold collapse occurs in cases in which redundant size of the AF^3^ or failure of the *transversus nasi* muscles action allows entry of air in the false nostrils that causes flaccidity of the AF and partial obstruction of the nasal cavities.^1^ Alar fold collapse usually causes a continuous vibrating/buzzing noise, usually present in both expiration and inspiration,^4^ associated with elevated expiratory nasopharyngeal pressures.^5^ This condition is a known cause for poor performance in equine athletes; AF collapse was diagnosed in 11% of Norwegian standardbreds examined for noise/exercise intolerance.^6^ After surgical resection of the AF in horses in which AF collapse was diagnosed, the expiratory nasopharyngeal pressure improved to reported normal levels.^5^

The diagnosis is usually made by placing a temporary mattress suture through the skin of each alar fold and tying it over the bridge of the nose while the horse exercises.^1^ This should alleviate the respiratory noise and improve performance. Before confirmation of the diagnosis, other causes of respiratory noise and exercise intolerance should be ruled out.

Complete surgical excision of redundant/flaccid AF is the treatment of choice when AF collapse is diagnosed.^3^, ^5^, ^7^ Traditionally, excision has been performed under general anesthesia (GA) through the natural orifice or via an incision on the lateral alae of the external nares.^1^ The resection is performed by sharp incision of the AF caudal to the alar cartilage on the lateral wall of the nasal cavity to the rostral end of the ventral conchae and a second incision along the medial attachment of the AF in a caudal direction until it joins the first incision.^7^ It has been reported that approximately 2 cm of the rostral ventral nasal conchae is removed with the AF and that profuse hemorrhage may occur when incising the cartilage. Hemostasis has traditionally been achieved by closure of the incision line with simple‐continuous sutures.^7^

Because this procedure has traditionally been performed under GA, there is an increased risk compared with procedures performed under standing sedation. This is particularly true for larger horse breeds and horses with concurrent musculoskeletal conditions, but GA also poses a risk even in young and healthy athletic horses.^8^ In addition, a standing procedure is cost efficient and, when the cost of GA is prohibitive, can be an important factor for allowing the client to pursue treatment. In recent years, several publications have described novel standing surgical techniques to perform procedures that were historically performed under GA.^2^, ^9^, ^10^, ^11^

The objective of this study was to describe a minimally invasive technique allowing complete resection of the AF via the natural orifice performed under sedation and local anesthesia (LA) with bipolar electrosurgical energy.

## 2 MATERIALS AND METHODS

### 2.1 Diagnosis

Medical records of standardbred racing trotters presented between April 2017 and August 2018 for noise and/or poor performance examination in which AF collapse had been diagnosed were reviewed and included in the study. The diagnosis was made during high‐speed exercise, either on a treadmill or while being driven on a track, and was characterized by the presence of noise with marked vibration of the AF at low and high speeds.

Improved performance (improved maximum speed on the high‐speed treadmill or when the horse ran the same track distance in a shorter time) and reduction of noise were noted when the AF were temporarily sutured up against the external nares or when the AF were sutured together by using a horizontal mattress USP 6 suture, as previously described.^1^ A complete upper airway endoscopy was also performed, when possible, on a high‐speed treadmill or on the track with an overground endoscope to determine whether there were any other associated conditions. An evaluation of the horse's demeanour was important to ensure that they would tolerate a standing surgical procedure.

### 2.2 Sedation protocol and local anesthesia

Consent forms were signed for all horses by the trainers. Horses were placed in stocks, sedated, and locally anesthetized. Sedation was achieved with acepromazine (0.02 mg/kg IV), detomidine (0.006‐0.008 mg/kg IV) first for the surgical site preparation, and LA. Then a second bolus of detomidine (0.006‐0.008 mg/kg IV) ± xylazine (0.15‐0.25 mg/kg IV) was administered just before the surgical procedure started. The horses also received flunixin meglumine (1.1 mg/kg IV) and procaine penicillin (22 000 IU/kg IM) preoperatively.

Bilateral infraorbital nerve blocks were performed in all the cases by using 5 to 10 mL mepivacaine per side. Additional anesthesia was administered in selected cases, consisting of subcutaneous infiltration of bupivacaine 5 mL at each incisive angle (corresponding with the caudal aspect of the AF); local anesthetic‐soaked tampons were also placed on the ventral/mucosal surface of the AF (one at a time to not block the airways).

### 2.3 Skin/mucosa preparation

**Figure 1:**
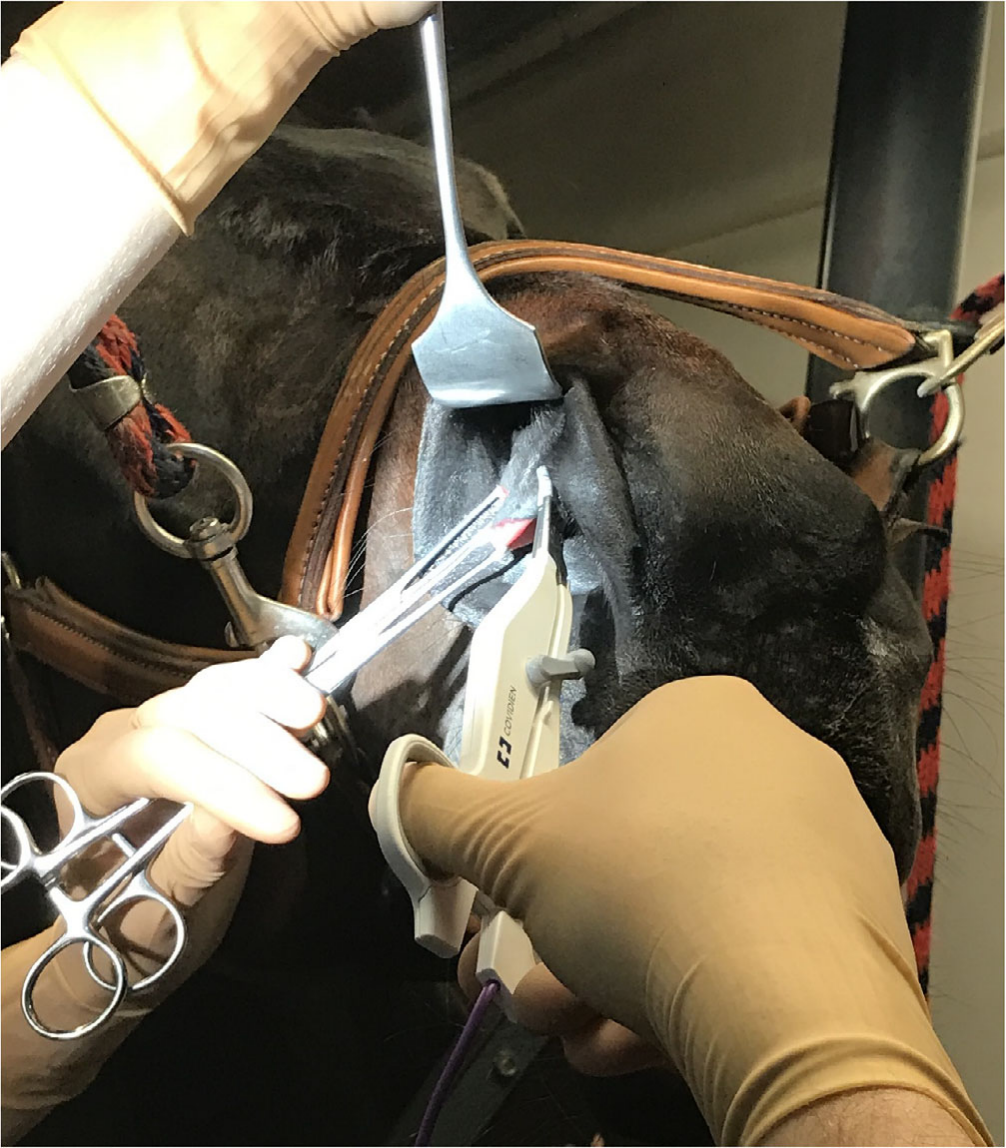
A Deaver or Richardson‐Kelly hand‐held retractor was inserted in the false nostril, and the AF was grasped and unrolled with two Allis tissue forceps by an assistant exposing the right AF. Transection was performed with the curved small‐jaw LigaSure device

The horse's head was elevated with a halter‐rope system (Figure 1). Skin and mucosal preparations of the surgical sites were then routinely performed with either diluted chlorhexidine or povidone iodine, and the disinfectant was rinsed with sterile saline.

### 2.4 Surgical technique

**Figure 2:**
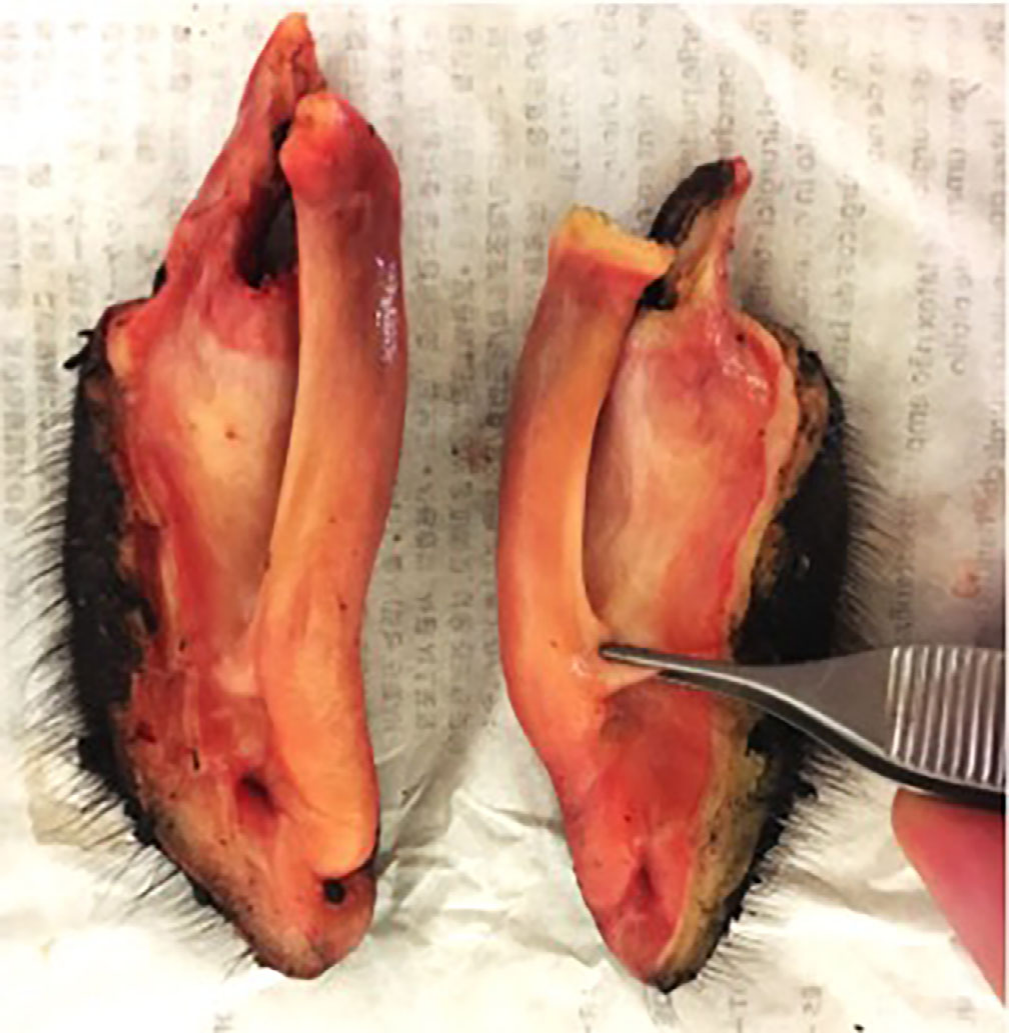
Ventral aspect of resected alar fold (rostral = bottom of the image). The forceps indicates the rostral aspect of the removed section of ventral conchal cartilage

A Deaver or Richardson‐Kelly hand‐held retractor was placed laterally in the false nostril to visualize the AF (Figure 1). The AF was grasped and unfolded by using two Allis tissue forceps. The fold was then resected under tension by using a curved, small‐jaw, open sealer/divider LigaSure device (setting 2/3; LigaSure™ Small Jaw Open Sealer/Divider, Medtronic order code LF1212). Specifically, the sealing/dividing instrument was “walked” from rostral‐to‐caudal, staying against the external nares for the dorsal incision line. The same procedure was then repeated on the ventroaxial aspect of the AF, keeping the instrument against the nasal cartilage. The entire AF (Figure 2), including some of the ventral conchal cartilage, was released when the transection lines met caudally. Two activations of the LigaSure device were often made caudally in the most vascular part of the AF before the blade was fired. The same procedure was repeated on the opposite AF. Finally, fucidine ointment was applied digitally on the surgery sites.

### 2.5 Postoperative care

Because the procedure was typically bloodless, the horses were immediately returned to their box stalls or discharged. When surgical site hemorrhage was noted, the horses were kept under monitoring until the bleeding discontinued.

Meloxicam 0.6 mg/kg was administered orally once daily for 3 days, then 0.3 mg/kg orally once daily for 2 days. The horses were fed moistened hay from the ground.

trainers were instructed to clean the/trainers were instructed to clean the nostrils daily with non‐woven swabs soaked in sterile saline. Local application of fucidine ointment daily for the first week was also recommended.

### 2.6 Postoperative exercise recommendations

The horses were typically box rested for 2 days. Hand walking of 10 minutes twice daily for 1 week was recommended. Then, the horses were allowed to return to walking exercise in the sulky or saddle for 1 week with paddock turn out. At 14 to 16 days postsurgery, the wound healing was assessed. If wound healing was satisfactory, jogging/trotting exercise was resumed, and no additional exercise restrictions were given.

### 2.7 Wound healing and performance/noise follow‐up

At 14 to 16 days postsurgery, evaluation of the wound healing was performed by a veterinarian to ensure that the horse was ready to resume unrestricted exercise and to be registered in races again. Trainer interviews were conducted within the first month postsurgery. Their subjective evaluations regarding respiratory noise progress and horse performance were recorded.

To evaluate the horses' performance progress objectively, race earnings for the three last races prior to surgery vs the three first races after surgery were compared. Racing times were considered less reliable, as potentially affected by race length and track conditions. Racing data were collected from the publicly available national racing database (http://www.travsport.no). To minimize the effect of time and other confounding factors on the surgery result and to give enough post‐surgery training time to the less mature racehorses, it was decided to stop the objective performance analysis at 8 months post‐surgery for every horse.

### 2.8 Statistical analysis

Race earnings for the last three races presurgery and for the first three races postsurgery were compared by using a Wilcoxon signed‐rank test. This nonparametric test was selected because the data were not normally distributed, the sample was small, and the test would incorporate the magnitude of the differences in earning. When a horse did not race either pre‐surgery or post‐surgery, it was excluded from the statistical analysis. When a horse registered in a race but did not earn any money, a zero was given as race earning value. Statistical significance was set at *P* < .05, and the statistical analysis was conducted in Stata SE/15 for Windows (StataCorp, College Station, Texas).

## 3 RESULTS

Eight horses (three geldings and five mares) met the inclusion criteria. They were 3 to 5 years of age (mean, 3.86), and their number of starts prior to surgery ranged from 0 to 36 (mean, 11.13).

### 3.1 Diagnosis

The diagnosis of AF collapse was made during high‐speed exercise on a treadmill (n = 3) or while being driven on a track (n = 8). Seven of eight horses had their AF sutured up and had improvement of noise and exercise tolerance (improved maximum speed). Three horses underwent an endoscopic examination on the high‐speed treadmill, and one horse had an overground endoscopic examination on the track. One horse was additionally found to have dorsal displacement of the soft palate (DDSP) and was treated simultaneously with laser palatoplasty. Another horse was found to have a mild degree of axial deviation of the aryepiglottic folds (ADAF). This was not treated at the time because it was considered too mild and likely related to the disturbance originating from the nares (AF collapse).

### 3.2 Surgical findings

Standing resection of the AF was successfully completed in all eight horses. All horses were adequately desensitized by the combination of local anesthetic techniques described. Some horses were sensitive/agitated when attempting to perform the infraorbital nerve blocks (3/8). Therefore, additional LA at the incisive angle and mucosal anesthetic tampons were added. Skin desensitisation was tested with thumb forceps, confirming achievement of a proper level of surgical analgesia before the first activation of the LigaSure device was made.

The surgical procedure, excluding skin/mucosa preparation and nerve blocks was short, and ranged from 20 to 30 minutes for the excision of both AF. The LigaSure open jaw sealer/divider occasionally slipped off rostrally when approaching the thick caudal part of the AF, causing a popping noise. The noise and vibration made some horses react, but this was not deemed a painful withdrawal response in the authors' opinion. To resolve this, the assistant surgeon applied additional rostral traction on the partially resected AF, and the surgeon was maintaining pressure caudally while activating the LigaSure jaws.

There was minimal (1/8) to no (7/8) bleeding recorded intraoperatively, and the procedure was well tolerated in all cases. For the horse who bled minimally postsurgery, the bleeding occurred when the head was returned to normal position while the horse was recovering from sedation and was still in the stocks. Arterial blood was noted to drip from one nostril, with initially one drop every 3 seconds. The horse was returned to its stall, and close monitoring was performed until the bleeding discontinued. The bleeding stopped without further intervention approximately 15 to 20 minutes postoperatively.

**Figure 3:**
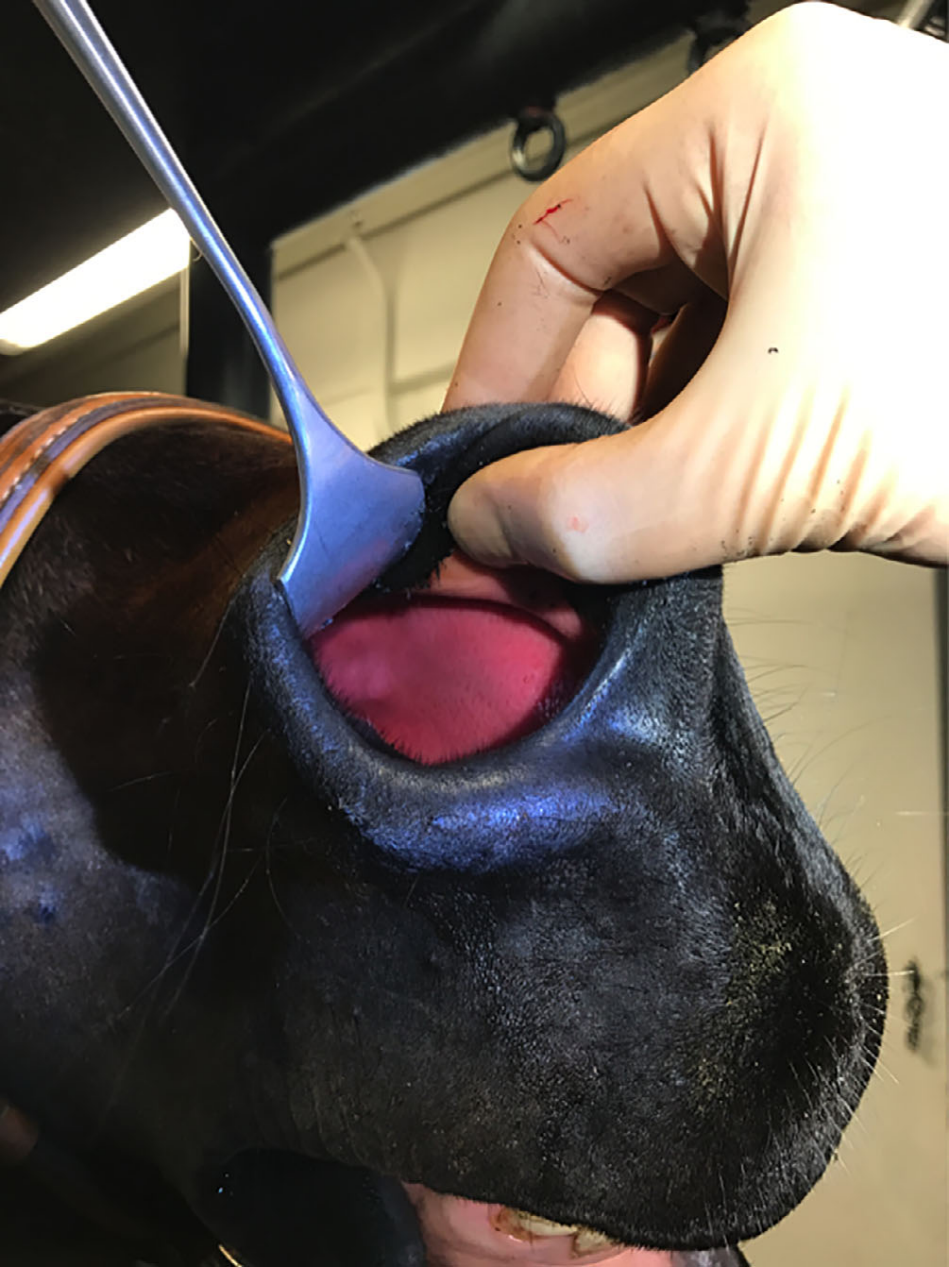
A large aperture is visible in the right nostril immediately postoperatively, and no bleeding is noted

A complete resection of the AF, including 3 to 5 cm of the ventral conchal cartilage, was achieved in all cases. A large aperture into the nasal passages was visible immediately postoperatively (Figure 3) in all nares.

### 3.3 Wound healing and performance/noise follow‐up

**Figure 4:**
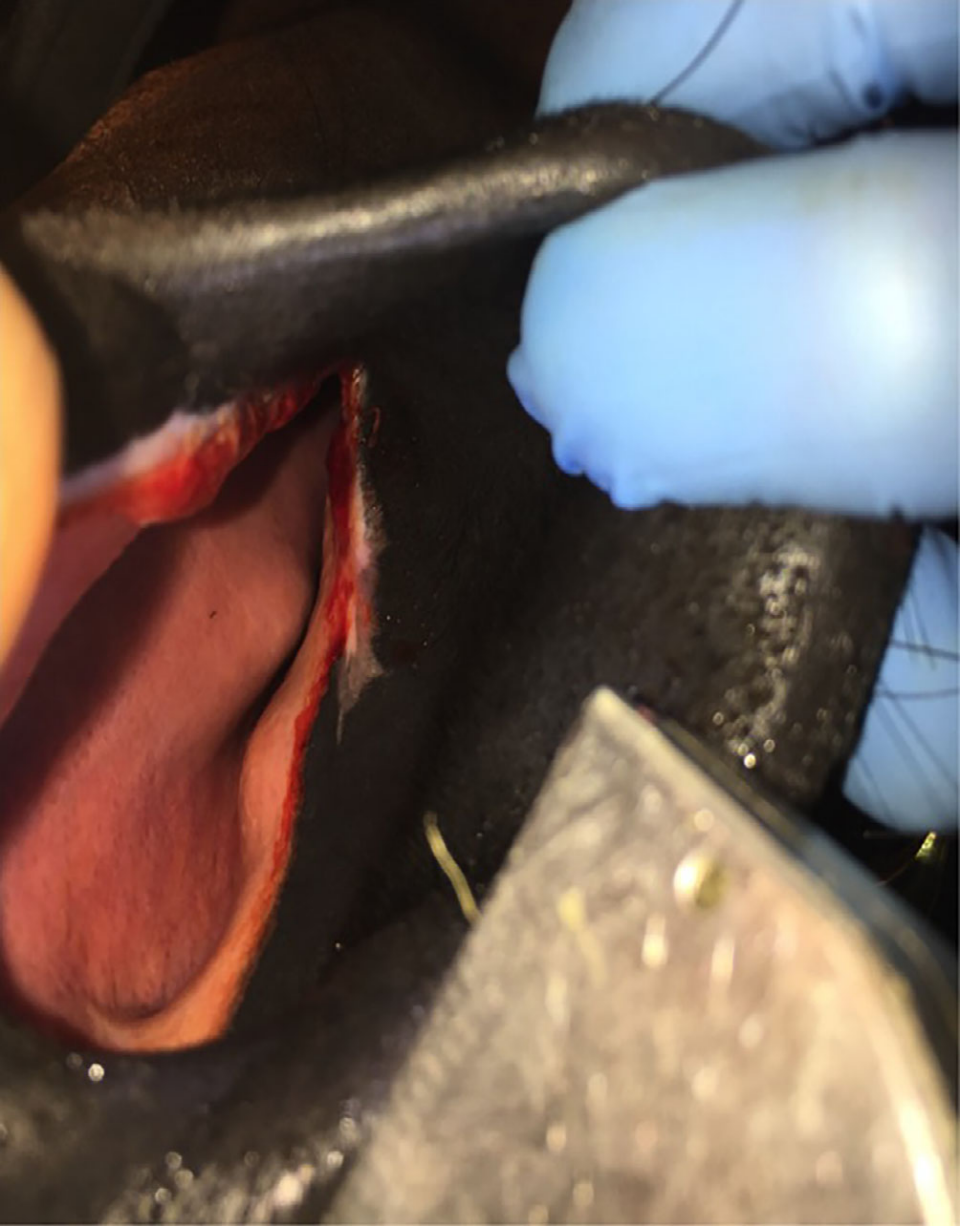
Left nostril at 3 weeks post‐surgery, after cleaning and crust removal

No complications were observed or reported during the post‐operative phase. None of the horses had any sign of nasal collapse, nasal cartilage necrosis, or infection. All horses had satisfactory subcrustaceous healing of the incision lines (Figure 4).

Noise reduction at exercise and improved performance were reported in all cases by the professional trainers. Return to training was achieved within 3 to 6 weeks after surgery in all cases. The mean and median times from surgery to first race were 120.5 days and 104 days, respectively, for the eight horses enrolled in the study. Two horses stood out with over 200 days from surgery to first race, but trainers reported that both horses had been sold shortly after surgery and that, in their opinion, this was the sole reason for the prolonged time until first race. When excluding these two horses, mean time from surgery to first race was 78.5 days.

**Figure 5:**
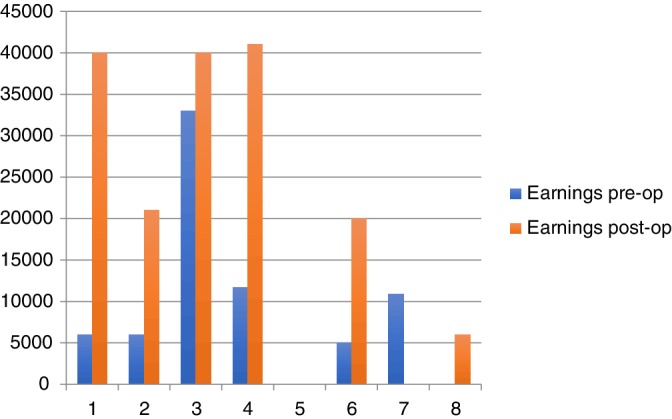
Earnings for each case in the three most recent races pre‐op (blue) and post‐op (red). Earnings are listed in Norwegian krones. Post‐op, post‐operatively; pre‐op, pre‐operatively

Improvement in race earnings was noted in six of eight (75%) cases (Figure 5). Horse 5 did not earn any money in the last three races prior to surgery or in the first three races after surgery. Two horses did not race pre‐surgery (horse 8) or post‐surgery (horse 7) and, therefore, were excluded from the statistical analysis. A difference in race earnings was noted post‐operatively compared with pre‐operatively by using the Wilcoxon signed‐rank test regarding the six horses that raced prior to and after surgery (*P* = .0345). Although the sample was small, this strongly supported the subjective opinion of the professional trainers involved in the study.

## 4 DISCUSSION

This study describes a novel minimally‐invasive surgical technique for the resection of redundant/unstable AF in the standing horse, its surgical outcome, and impact on the racing performance of eight trotter racehorses. Improvement in noise and performance was recorded and was associated with significant increase in prize money post‐surgery. The risk associated with GA was bypassed, and the procedure was well tolerated in all cases. The surgery was performed via the natural orifice to minimize rehabilitation time and potential complications. Bipolar electrosurgical energy was selected to resect AF, allowing removal of more tissue as previously described^7^ and ensuring minimal to no bleeding, without wound healing complications.

Decrease in respiratory noise and an improvement in exercise tolerance has been reported in 71% of the horses that underwent AF resection for the treatment of AF collapse.^3^ In this current case series, 100% of the horses had a reduction in respiratory noise and improved performance, as reported by the trainers. This information is subjective and may be biased. Professional trainers, although very attentive to the health and performance of their horses, may be inclined to give positive feedback to justify the decision to perform surgery and expenses to their clients. Therefore, the authors decided to objectively evaluate the surgery results by comparing earnings of three races prior to and after surgery. Improvement in earnings was noted in six of eight cases (75%), and this was significant for the whole group (*P* = .0345), strongly supporting the subjective opinion of the professional trainers involved in this study. These results provide evidence that the novel technique described here produces outcomes comparable to the traditional surgical technique.

The importance of confirming the presence of AF collapse before surgery cannot be overemphasized because it is key to obtaining satisfactory results with any AF resection technique. Despite the fact that the diagnosis was confirmed in all cases by direct observation (presence of noise with marked vibration of the AF at low and high speed) in our study, two horses did not improve their performance post‐surgery. These included one horse that had no earning before and after surgery and one horse that had a prize money reduction post‐surgery. During the diagnostic phase, seven of eight cases objectively improved their performance after the AF were temporarily sutured; this confirmed the direct link between AF collapse and reduced performance.^3^, ^7^ Dynamic endoscopy was performed in only four of eight cases because of the limitations inherent to a clinical study investigating client‐owned horses. One horse that did not have improved earnings post‐surgery did not have endoscopy performed at exercise. It is likely that this case presented additional undiagnosed respiratory issues that significantly affected performance. Associated upper airway conditions, either secondary to AF collapse or isolated, are not uncommon and are detected during dynamic upper airway endoscopy.^6^ Although earnings post‐surgery significantly improved for the whole group, in the authors' experience and in light of previous studies,^5^, ^6^, ^12^ a full dynamic endoscopic examination should be performed in all cases in which AF collapse is diagnosed to ensure that no additional problems would require attention.

Among the two horses in which other upper respiratory tract disorders were diagnosed during dynamic endoscopy, the horse diagnosed with a concomitant DDSP underwent a laser palatoplasty^13^ under standing sedation and LA in conjunction with the AF resection. The trainer was advised to first address the AF collapse by performing the standing resection and then to re‐evaluate the horse 3 to 4 weeks later to determine whether the DDSP would still be present. This approach was declined, and the trainer requested a laser palatoplasty on the same day to address the DDSP, whether primary or secondary to AF collapse. Being able to perform AF resection and laser palatoplasty under standing sedation enabled the authors to perform both treatments in an effective way, minimizing risks associated with GA and making the procedure cost effective for this trainer. Another horse was diagnosed with mild ADAF, but this was thought to be influenced by the AF collapse. The trainer followed the recommendations to re‐evaluate the horse 3 to 4 weeks post‐AF resection. This horse performed well after the AF resection, so the ADAF was not addressed.

When the diagnosis is appropriate and AF collapse is the only cause of poor performance, failure post‐surgery can be due to an incomplete resection of the AF, mainly in its caudal region. Although this did not occur in our case series, the surgical site should be scrutinized in these cases. This is why some surgeons prefer to open the external nares to ensure that complete resection of the AF is performed.^5^ Additional advantages include excellent visibility allowing precise removal of all redundant tissue and cartilage to create a streamlined passageway (funnel) for airflow and primary closure. This invasive approach was not considered necessary in the authors' opinion. The use of a good retractor and of the curved small‐jaw LigaSure device allowed complete exposure and complete caudal reach, as was illustrated by the 3 to 5 cm of ventral conchal cartilage that was resected in addition to the entire AF in all cases. As a result, a wide opening into the ventral meatus was generated without incising into the external nares. Surgery performed via the natural orifices carry a significant advantage over open approaches because it reduces the post‐operative convalesce and potential wound complications.^14^ For cases with external nares flaccidity, incision and scaring of the external nares can be beneficial, but this condition is a separate entity from the AF collapse.^15^

Some surgeons have suggested suturing the tissue to reduce bleeding and to improve healing.^1^ The LigaSure device used in our study allowed either complete hemostasis or minimal intraoperative bleeding, which is a vast improvement compared with surgeries performed under GA. This was likely due to keeping the head positioned above the heart, reducing blood pressure, and to electrosurgical energy that allowed coagulation of small to medium‐sized blood vessels. To reduce the surgical trauma and time and because hemorrhage was none to minimal, suturing of the incised tissue was not considered essential in our study and led to uncomplicated healing of the incision lines under a crust.

It is important to note that, in the eight horses treated in this study (16 surgery sites), the use of this curved small‐jaw LigaSure device (setting 2/3) did not lead to any wound‐healing complication, such as ventral conchal cartilage necrosis. The authors think it is essential, however, not to overactivate the LigaSure device. No more than two activations per bite was required to achieve perfect hemostasis. Although cartilage necrosis was not observed in our study, a larger case series may shed more light on this potential issue that would surely delay wound healing.

In a study by Hawkins and colleagues,^3^ the mean time to first start postsurgery was 118 days, while in our case series the mean time to first race, excluding two horses with prolonged time to racing for unrelated reasons, was 78.5 days (range, 28‐251). The median time for all eight horses was 104 days from surgery to first race. This is suggestive of a reduced convalescence with this novel surgical approach.

To the best of the authors' knowledge, AF resection has not been reported in standing horses, and the use of an electrosurgical sealing device, specifically the curved small‐jaw, open sealer/divider LigaSure device, has not previously been described for this procedure. Owners/trainers were interested in this standing approach because the risk and cost associated with GA was avoided. A selection of local anesthetic techniques completely anesthetized the surgical site, allowing completion of the surgical procedure in all cases without any movement. It is unclear at this stage whether more than just a bilateral infraorbital nerve block is required, and additional research work is required to clarify this.

Acepromazine was selected to reduce intraoperative bleeding because of its hypotensive effect.^16^ The xylazine and detomidine combination has not been described during standing perioperative sedation protocols in the horse. It was selected to potentiate the effects of both sedatives because they have different affinity for the α‐2A and α‐2C receptors. Stimulation of these receptor subtypes is responsible for sedation and analgesia.^17^ Butorphanol was not selected because of the undesirable jerking head movement that has sometimes been noted when it is used, which is disruptive when head surgery is being performed.^18^

In conclusion, the preliminary results of this study provide evidence that standing AF resection with bipolar electrosurgical energy offered a good alternative to the traditional surgical approaches performed under GA. Adding additional cases and long‐term follow‐up would provide stronger evidence to support this approach in racehorses.

## CONFLICT OF INTEREST

The authors declare no conflicts of interest.
